# The holistic effects of medical cannabis compared to opioids on pain experience in Finnish patients with chronic pain

**DOI:** 10.1186/s42238-023-00207-7

**Published:** 2023-11-09

**Authors:** Jussi Jylkkä, Aleksi Hupli, Aleksandra Nikolaeva, Sandra Alanen, Anna Erika Back, Sara Lindqvist, Andreas Krabbe, Maya Lavie-Ajayi, Oskari Kantonen

**Affiliations:** 1 Department of Psychology, Åbo Akademi University, Turku, Finland; 2 Emerging Technologies Lab, Faculty of Social Sciences, Tampere University, Tampere, Finland; 3 Turku Brain and Mind Center, Faculty of Medicine, University of Turku, Turku, Finland; 4 Gender Studies Program, Ben-Gurion University of the Negev, Be’er Sheva, Israel; 5 Department of Perioperative Services, Intensive Care and Pain Medicine, Turku University Hospital, University of Turku, Turku, Finland

**Keywords:** Chronic pain, Medical cannabis, Opioids, Experience, Consciousness, Psychoactive effects, Wellbeing, Functionality, Mood, Negative side effects

## Abstract

**Background:**

Medical cannabis (MC) is increasingly used for chronic pain, but it is unclear how it aids in pain management. Previous literature suggests that MC could holistically alter the pain experience instead of only targeting pain intensity. However, this hypothesis has not been previously systematically tested.

**Method:**

A retrospective internet survey was used in a sample of Finnish chronic pain patients (40 MC users and 161 opioid users). The patients evaluated statements describing positive and negative phenomenological effects of the medicine. The two groups were propensity score matched to control for possible confounding factors.

**Results:**

Exploratory factor analysis revealed three experience factors: Negative Side Effects, Positive Holistic Effects, and Positive Emotional Effects. The MC group (matched *n* = 39) received higher scores than the opioid group (matched *n* = 39) in Positive Emotional Effects with large effect size (Rank-Biserial Correlation RBC = .71, *p* < .001), and in Holistic Positive Effects with medium effect size (RBC = .47, *p* < .001), with no difference in Negative Side Effects (*p* = .13). MC and opioids were perceived as equally efficacious in reducing pain intensity. Ratings of individual statements were exploratively examined in a post hoc analysis.

**Conclusion:**

MC and opioids were perceived to be equally efficacious in reducing pain intensity, but MC additionally positively affected broader pain-related factors such as emotion, functionality, and overall sense of wellbeing. This supports the hypothesis that MC alleviates pain through holistically altering the pain experience.

**Supplementary Information:**

The online version contains supplementary material available at 10.1186/s42238-023-00207-7.

## Background

Chronic pain refers to pain that has pertained for at least three consecutive months. In the European Union, 19% of adults suffer from chronic pain that has lasted for more than six months (Breivik et al. 2006). In Finland, 35% of adults have suffered from pain of at least three months duration, and the prevalence of daily chronic pain is 14% (Mäntyselkä et al. 2003). Both opioids and medical cannabis (MC) are commonly used for pain alleviation (Schlag et al. 2021; Wertheimer et al. 2021). Opioids are known to efficiently alleviate pain both acutely and chronically (Meske et al. 2018), but they have several adverse side effects, some of them potentially fatal (Paul et al. 2021). While the side effects of MC are mainly non-severe (Deshpande et al. 2015; Ware et al. 2015), its efficacy is disputed and mechanisms of action lack clarity (Fisher et al. 2021; Häuser et al. 2018). These unclarities could stem from many reasons, including the great variety of cannabis based medical products and variants of cannabis plants (Russo & Marcu 2017; Schlag et al. 2021), as well as the complexity of the molecular targets of different cannabinoids (Mlost et al. 2020; Morales et al. 2017). Another possible reason for the inconsistencies in previous research is the complex effects of cannabis on the psyche, which is our focus in the present study.

Previous research has mainly focused on the analgesic effect of MC (i.e., its capacity to reduce pain intensity), but pain sensation is a multifaceted experience involving more than nociception (St. John Smith 2018). It is well-known that cannabis can alter the state of consciousness, but not much attention has been paid to the question whether this could constitute a part of the therapeutic effect of MC on pain. In a previous qualitative interview study of seriously and terminally ill patients in California (Chapkis 2007), the psychoactive effects of MC were associated with feelings of wellness, increased acceptance of the pain, being able to ignore the pain and do other things, uplifting of the spirit, increased focus, as well as psychological and spiritual insights. On the other hand, unwanted side-effects, such as disturbed memory processes, tolerance, and dependence were also reported. Another qualitative study with chronic pain patients from an Israeli pain clinic (Lavie-Ajayi & Shvartzman 2019) found that MC facilitated the development of a different bodily subjectivity, described as a “sigh of relief”, a sense of relaxation and serenity that allows the patients an opportunity to unload some of the tension experienced by the constant fight against the pain. In addition, the use of MC was also described by many patients as having a holistic effect that enabled them to function better in their daily lives, including increased ability to sleep, focus, and function (ibid.).

In addition to these qualitative studies, there is quantitative research supporting the notion that MC has therapeutically relevant positive effects beyond pain intensity per se. A systematic review of placebo-controlled studies on the effects of cannabis on acute experimentally induced pain in healthy participants found that cannabis did not affect perceived pain intensity, but instead made the pain feel less unpleasant and more tolerable (De Vita et al. 2018). Further, a cross-sectional study on chronic pain patients found that MC users experienced substantially less depression and anxiety than opioid users (Feingold et al. 2017), suggesting that MC may have therapeutic effects in chronic pain patients over and above reducing pain intensity. The use of MC in chronic pain has also been associated with improved physical and social functioning and overall quality of life (Haroutounian et al. 2016; Pritchett et al. 2022; Vigil et al. 2017), as well as improvements in mental health and anxiety (Safakish et al. 2020). On the neurocognitive level the pain-alleviating effects of MC have been associated with changes in connectivity between brain regions associated with emotional regulation and lower somatosensory areas, suggesting that cognitive-emotional modulation may mediate the effects of MC on pain (Weizman et al. 2018). There is also evidence that THC reduces the perceived unpleasantness of experimentally induced pain, correlated with amygdala activity and reduced sensory-limbic functional connectivity (Lee et al. 2013), leading these authors to conclude that the “dissociative” effects of THC are relevant to pain relief. Importantly, a recent naturalistic study utilizing data from 1,882 users of a medical cannabis treatment tracking app directly assessed the association between feeling “high” and experiencing therapeutic effects (Stith et al. 2023). Overall, feeling high was associated with symptom relief across most of the patient subgroups, including those who used MC for pain relief, but feeling high also predicted more negative side effects (ibid.).

The previous research thus supports the hypothesis that MC may exert its therapeutic effect on pain through altering the pain experience in a more holistic way than traditional analgesics. That is, in addition to having an antinociceptive effect (i.e., removing pain or lessening its intensity), MC may affect the pain experience more broadly, influencing factors such as mood and emotion, pain tolerance, functionality, and overall well-being. On this account, the consciousness-altering psychoactive effects of MC could be a part of its therapeutic mechanism, instead of merely negative side effects. This hypothesis has not, however, been systematically tested. In this preregistered retrospective survey study, we recruited Finnish chronic pain patients who use either opioids or MC for their pain (preregistration at https://osf.io/txaph). We asked the patients to rate how they experienced the effects of the medicine (opioids or MC), using a questionnaire. The preregistered hypothesis was that whereas opioids mainly affect the intensity of the pain, MC affects the pain experience in a more holistic way, affecting factors such as functionality, emotion and mood, and mindfulness. Our focus was on the perceived effects of MC, while the opioid group mainly served as controls. Neither MC nor opioids are first-line treatments for chronic pain, but both are commonly used when other treatments fail to provide sufficient pain relief. This could render the opioid and MC users similar in relevant background characteristics such as severity of pain and the underlying diagnosis, enabling better control of confounding factors.

## Materials and methods

A cross-sectional survey study was employed on Finnish patients suffering from chronic pain who used MC or opioids for pain management. We devised a set of 45 experience questions to assess the overall perceived effects of the medicine, inspired both by the previous qualitative studies (Chapkis 2007; Lavie-Ajayi & Shvartzman 2019) as well as previous questionnaires such as the Brief Pain Inventory (Cleeland & Ryan 1994) and the Five Facet Mindfulness Questionnaire (Baer et al. 2006). The questions included items tapping on emotional factors such as “The medicine makes me more relaxed” or “improves my mood”; functional aspects such as “helps me to take care of myself” or “enables me to do the things I like”; mindfulness-related questions such as “enables me to feel the pain without reacting to it”, as well as holistic factors such as “has enabled me to gain a sense of normality in my life” or “has improved my life quality”. All the questions were answered on a seven-point Likert scale, where 1 indicates “Completely of the opposite opinion” and 7 “Completely of the same opinion”. The points were labeled, the midpoint (4) as “Neither of the opposite nor the same opinion”. All the questions are listed in Appendix A.

Information about the pain condition was gathered with the Chronic Pain Questionnaire (Pfizer 2021); psychiatric conditions were probed with the DSM-5 Self-Rated Level 1 Cross-Cutting Symptom Measure—Adult (Bravo et al. 2018); and severity of dependence was assessed with the Severity of Dependence Scale (Rush et al. 2003). The diagnosis underlying the pain condition was assessed with a multiple-choice question and the answers were recoded based on the open reports about the type of pain as well as the reported ICD-10 codes into the following categories: 1) Chronic musculoskeletal pain; 2) Chronic neuropathic pain; 3) Other specified chronic pain (e.g., fibromyalgia and type I complex regional pain syndrome, CRPS) or chronic pain of unknown etiology; 4) Chronic cancer-related pain; 5) Chronic visceral pain; and 6) Chronic headache or orofacial pain.

Opioid and cannabis use was probed by asking whether the patient uses these substances for pain management (yes / no), what is the weekly use frequency, average dose, type of the medicine, whether they had used the medicine during the last week, and prescription status (yes / never / discontinued). The options for the type of opioid were “weak (e.g., codeine, tramadol)”, “medium (e.g., burprenorphine)”, and “strong (e.g., fentanyl, hydromorphone, methadone, morphine, oxycodone)”. For MC, the options were “Bedrocan”, “Bediol”, “Sativex”, “Don’t know”, and “Other, please specify (e.g., what variety)”. Additionally, in the case of MC, the THC/CBD content was probed with the options “Balanced (50/50)”, “THC dominant”, “CBD dominant”, and “Don’t know”. The whole questionnaire is included as Appendix B.

The survey was distributed through email lists of Finnish chronic pain patient organizations, the Finnish Medical Cannabis Association, social media (e.g., Twitter and Facebook), as well as with flyers spread to the local smartshops selling cannabis-related equipment. The survey link led to a landing page including information about the study and eligibility criteria, which was that the patient suffers from chronic pain and uses opioids or medical cannabis to treat it. The eligibility was checked by examining the responses to the survey questions about pain duration (minimum three months), description of the pain diagnosis and symptomology, and types of medication used to treat it. Recreational users of cannabis or opioids and those who use the substances to treat other types of symptoms than chronic pain were explicitly asked not to partake in the study. To determine how to sum up the experience questions, we utilized exploratory factor analysis (EFA), following the recommendations of Worthington & Whitaker (2016) and Howard (2016). We used exploratory factor analysis and principal axis factoring (PAF) and the rotation method Promax (with kappa = 4) which allows for correlated factors. Problematic items were deleted following the 0.40—0.30—0.20 rule: an item should have at least 0.40 loading on the primary factor, max 0.30 loading on any secondary factors (i.e., “cross-loading”), and minimum difference between the primary and secondary loadings should be 0.20 (Howard 2016).

To account for background characteristics that may confound the results, we used propensity score matching (PSM) (for a general introduction, see Harder et al. 2010) on the following covariates: age, gender, type of pain, duration of pain, diagnosis (yes/no), as well as income and education level. Psychiatric variables such as anxiety and depression were not used as covariates, as they could be affected by the treatment (Feingold et al. 2017). The matching was conducted with the Matchit package (Ho et al. 2011) under R version 4.2.2 (R Core Team 2021), using the nearest neighbor method with 1:1 matching and logistic regression.

The preregistered analyses method for group comparisons was t-tests, but Mann–Whitney U-tests were used instead due to normality violations. Multiple comparisons were corrected by Bonferroni correction. Rank-Biserial Correlation was used as a non-parametric estimate of standardized effect size. Additionally, to see whether there are interactions between the experience factors and group, repeated ANOVAs were used.

In addition to frequentist p-values, Bayes factors were calculated to estimate the strength of evidence for the alternative in contrast to the null hypothesis. The Bayes factor BF_10_ indicates the likelihood of the observed data if the alternative hypothesis holds, in proportion to its likelihood if the null hypothesis is true. For example, if BF_10_ = 3, the data is three times more likely if the alternative hypothesis H1 is true. The BF_10_ is interpreted as follows: > 100 Extreme evidence for H1; 30 – 100 Very strong evidence for H1; 10 – 30 Strong evidence for H1; 3 – 10 Moderate evidence for H1; 1 – 3 Anecdotal evidence for H1; 1 No evidence. Values below one indicate evidence for the null hypothesis, for example BF10 = 1/4 indicates moderate evidence for the null hypothesis (Jeffreys 1961).

The study was approved by the ethical council for psychology and logopedics at Åbo Akademi University, Finland. The testing was conducted by using an anonymous online survey that was distributed through patient organizations, social media and other social networks (e.g., patient groups). No compensation was given for participation.

## Results

The participants (*N* = 201) were divided into two groups, MC users (*n* = 40) and opioid users (*n* = 161), based on which medicine they selected for evaluation in the experience questions. In the MC group, 18 (45%) reported also using opioids for their pain, and in the opioid group seven (4.3%) reported also using MC for their pain. Of the MC users, 29 (73%) reported having used MC during the last week, and in the opioid group 149 (93%) had used opioids during the last week. Moreover, of the MC users, 12 (30%) reported having used opioids during the last week, and of the opioid users two (1.2%) reported having used MC during the last week. In the opioid group 157 (99%) had an active prescription for the medicine and only one person had a discontinued prescription (prescription information was missing from three participants). By contrast, in the MC group only 10/40 (25%) had an active prescription, 11/40 (27.5%) had a discontinued prescription, and 17/40 (42.5%) had never had a prescription (prescription information was missing from two participants). We included in the study even the MC patients without prescription, given the substantial practical difficulties in getting prescription for MC in Finland (see Discussion). In the MC group, 37/40 (93%) had a diagnosis and in the opioid group 159/161 (99%); this difference was significant in frequentist analyses but not supported by the Bayesian analysis (cross-tabulation BF_10_ = 1/1.6, χ^2^ = 5.17, *p* = 0.023). Demographic information and pain type with tests of group differences are described in Table 1.

**Table 1:** Background information with between-group differences. Group is determined by the medicine the participants evaluated in the experience questions

	**MC**		**Opioid**			
	***MC***	***SD***	***M***	***SD***	***p***	**BF**_**10**_
Age (years)	43.00	9.83	47.76	10.79	0.012	3.47^a^
Income [1–5]	1.88	1.09	2.13	1.06	0.120	1/3.57
Education [1–7]	2.85	1.44	3.46	1.72	0.055	1/2.13
Pain duration (months)	165.36	105.12	163.58	108.31	0.840	1/5.20
Average pain over the past week (CPQ) [0–10]	5.98	2.21	6.72	1.49	0.110	1/2.17
Interference of pain with activities (CPQ) [0–10]	5.23	2.42	6.04	1.70	0.082	1/1.85
Depression score (DSM5) [0–4]	2.25	1.06	2.47	0.96	0.310	1/4.35
Anxiety score (DSM5) [0–4]	1.65	1.08	1.71	1.11	0.840	1/5.26
Severity of dependence (SDS) [0–15]	2.63	2.63	3.60	2.81	0.020	1/1.92
**Gender**						
	**%**	***n***	**%**	***n***	***p***	**BF**_**10**_
Male	70	28	19	30	< .001	> 100^b^
Female	28	11	78	126	< .001	> 100
Other	3	1	2	4	1.00	1/2.44
**Pain type**						
	**%**	***n***	**%**	***n***	***p***	**BF**_**10**_
Musculoskeletal	53	21	59	95	0.46	1/5.26^b^
Neuropathic	60	24	72	116	0.14	1/2.17
Cancer-related	3	1	2	4	1.00	1/2.44
Visceral	30	12	29	47	0.92	1/6.25
Head	10	4	6	10	0.40	1/6.25
Other	60	24	63	101	0.75	1/6.67

^a^Student t-test was used for age, Mann–Whitney U-test for all other continuous variables

^b^Cross-tabulations were used for categorical variables

Income was assessed with a 5-point scale from “Substantially below average” (1) through “Average” (3) to “Substantially above average” (5)

Education refers to highest completed degree: 1 = Elementary school, 2 = Vocational school, 3 = High school, 4 = University of applied sciences, 5 = Lower university degree (bachelor), 6 = Higher university degree (master), 7 = Doctoral degree

*MC* Medical Cannabis, *DSM5* Self-Rated Cross-Cutting Symptom Measure, *CPQ* Chronic Pain Questionnaire

Theoretical range for the variable in question is indicated in square brackets

In the opioid group, 77 (48%) reported using a weak opioid (e.g., codeine or tramadol), 32 (20%) used a medium strength opioid (e.g., buprenorphine), and 50 (31%) used strong opioids (e.g., fentanyl or oxycodone), and data was missing for 2 cases. 149 (93%) reported using opioids within the last week, and total lifetime duration of opioid use was on average 7.12 years (SD = 6.28). Opioids were used in this group on average 9.50 times a week (SD = 6.59). However, weekly use data may not be reliable, as several patients reported using the medicine “continuously” (e.g., as transdermal patch) and these cases were coded as “7 times a week”. The average Morphine Equivalent Dose (MED) was 41.34 mg (SD = 37.67, range = 2 – 210), however missingness was *n* = 61 due to limited details about the type of opioid and use frequency which made it impossible to reliably calculate the MED for these participants.

In the MC group, 18 (45%) reported using Bedrocan, 1 (2.5%) used Bediol, 9 (22.5%) used some other type of specified cannabis flower, and 10 (25%) reported that they did not know the type of the cannabis they used. 29 (73%) had used MC during the last week. As to the ratio of THC and CBD, 24 (60%) reported that their medicine was THC-dominant, 7 (18%) reported balanced, and 2 (5%) reported CBD-dominant, whereas 5 (13%) did not know this information. Lifetime duration of MC use was on average 7.46 years (SD = 5.87) and average weekly use was 19.76 times (SD = 19.36). Average single dose was 0.65 g (SD = 1.01; range 0.01—5 g). As a method of use, 16 (40%) reported vaporizing, 12 (30%) reported smoking, 6 (15%) reported eating, and 4 (10%) reported “other” (e.g., tea, oil, or combined methods).

### Factor analysis of the pain experience questions

The Kaiser–Meyer–Olkin (KMO) measure of sampling adequacy was excellent (0.93) and Bartlett’s test of sphericity was passed (χ^2^ = 7519, df = 990, *p* < 0.001). Based on the scree plot, four factors were extracted. Before proceeding in the analysis, we examined the factor structure for face validity. The fourth factor included only two items (i.e., item 34, “The medicine causes hallucinations” and item 36, “The medicine makes me paranoid”) and was thus omitted. A three-factor analysis was then run without these two questions and the model explained 56% of the variance. Next, 13 problematic items were deleted due to violating the 0.40—0.30—0.20 rule (see Method). Reliabilities in terms of Cronbach’s alphas were 0.93, 0.87, and 0.90 for the three factors respectively, indicating good-to-excellent reliability. The final 30 items and their factor loadings are presented in Table 2.

**Table 2:** Final items and extracted factors, with problematic items deleted

		**Factor loadings**		
**Item**	**Question ("The medicine…")**	**1 Negative side effects**	**2 Positive Emotional**	**3 Positive Holistic**
35	makes my thoughts blurry	**0.89**	0.03	0.06
13	makes it more difficult to focus on my environment and what happens around me	**0.84**	0.06	-0.01
19	makes me feel powerless (lack energy)	**0.82**	0.20	-0.13
33	makes it hard to focus	**0.80**	-0.02	0.01
39	makes me anxious	**0.77**	0.08	-0.05
28	impairs my memory	**0.75**	-0.05	0.17
3	hinders me from being the best version of myself	**0.70**	-0.13	0.14
27	makes me socially withdrawn	**0.70**	0.01	-0.08
16	makes me dizzy	**0.70**	0.20	-0.07
45	lowers my mood	**0.68**	-0.11	-0.02
21	causes me negative physical symptoms	**0.67**	-0.15	0.18
23	makes me nauseous	**0.60**	-0.11	0.05
26	worsens my sleep quality	**0.52**	-0.24	0.23
43	makes me less anxious	-0.03	**0.89**	-0.16
40	improves my mood	-0.02	**0.88**	-0.04
38	makes me more relaxed	0.14	**0.85**	-0.02
42	helps me feel more emotionally stable	-0.11	**0.81**	-0.09
24	makes it easier to breathe	0.18	**0.61**	0.08
22	enables me to sleep better	0.13	**0.55**	0.17
14	enables me to feel the pain without reacting to it	0.12	**0.52**	0.00
9	lessens the intensity of the pain	0.10	-0.23	**1.03**
8	makes the pain more tolerable	0.08	-0.19	**0.96**
11	enables me to focus on other things beside the pain	0.02	-0.05	**0.87**
4	has improved my life quality	-0.14	-0.03	**0.79**
5	has enabled me to regain a sense of control over my life	-0.02	0.21	**0.65**
2	has enabled me to gain a sense of normality in my life	0.08	0.27	**0.64**
10	eliminates the pain	0.11	0.10	**0.55**
12	enables me to pay more attention to sensations (like wind on my cheek, clock ticking, objects’s textures and patterns)	0.19	0.26	**0.54**
15	helps me to take care of myself	-0.13	0.26	**0.54**
17	enables me to do the things I like	-0.13	0.30	**0.53**

We determined that the first factor (13 items) included negative side effects, including cognitive disturbances, emotional problems, as well as functional and somatic problems. This factor was titled “Negative Side Effects”. The second factor (7 items) mainly consisted of positive emotional effects and was titled “Positive Emotional Effects”. The third factor (10 items) consisted of a broader range of positive effects: The factor was driven by the items 9 and 8 about pain intensity and pain tolerance, but also consisted of holistic and functional items such as “Focus on other things” (item 11), “Improves life quality” (item 4), “Regain normality” (item 2), “Regain control” (item 5), “Take care of myself” (item 15), and “Attention to sensation” (item 12) (see Table 2). This factor was titled “Positive Holistic Effects”. The questions were averaged to gain factor scores.

### Group comparisons

**Fig. 1:**
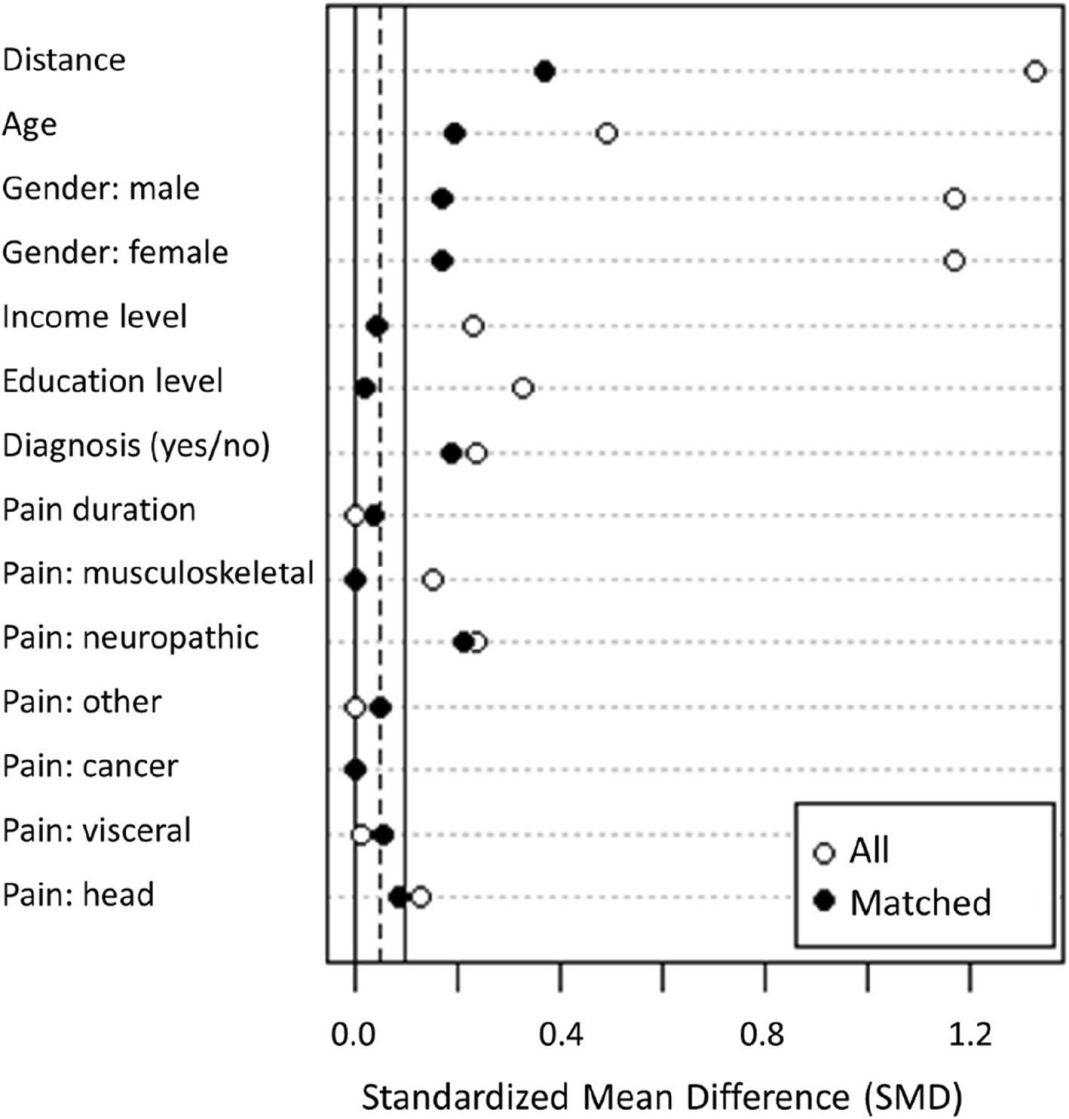
Comparison of the matched vs. non-matched groups on the covariates. Although no ideal match was attained on all covariates, the match between the groups was substantially improved. *Note*: The standardized mean difference (SMD) indicates the extent to which the groups differ on a given covariate; smaller values are desired. The highlighted vertical line at .1 indicates the recommended maximum SMD. For information regarding how the background variables were assessed, see Table 1

Before comparing the perceived effects of the medicine between the groups, matching was conducted for the relevant covariates (see Methods). Data was missing about the duration of pain for ten participants (one in MC group and nine in the opioid group) and was imputed by the respective group means. Moreover, education data was missing from six participants in the opioid group and was imputed with the respective group mode two (corresponding to vocational school). We removed cases that reported as gender “other” or did not disclose this information (five in the opioid group and one in the MC group) due to an unreliably small number of observations. The matched data (*n* = 39 in both groups) showed overall better balance than the unmatched sample, although some of the covariates were still above the recommended threshold value of 0.1 (see Fig. 1) (Stuart, Lee, & Leacy 2013). However, since no matching method yielded perfect balance and all the standardized mean differences for individual predictors were < 0.2, we proceeded to the main analysis.

**Fig. 2:**
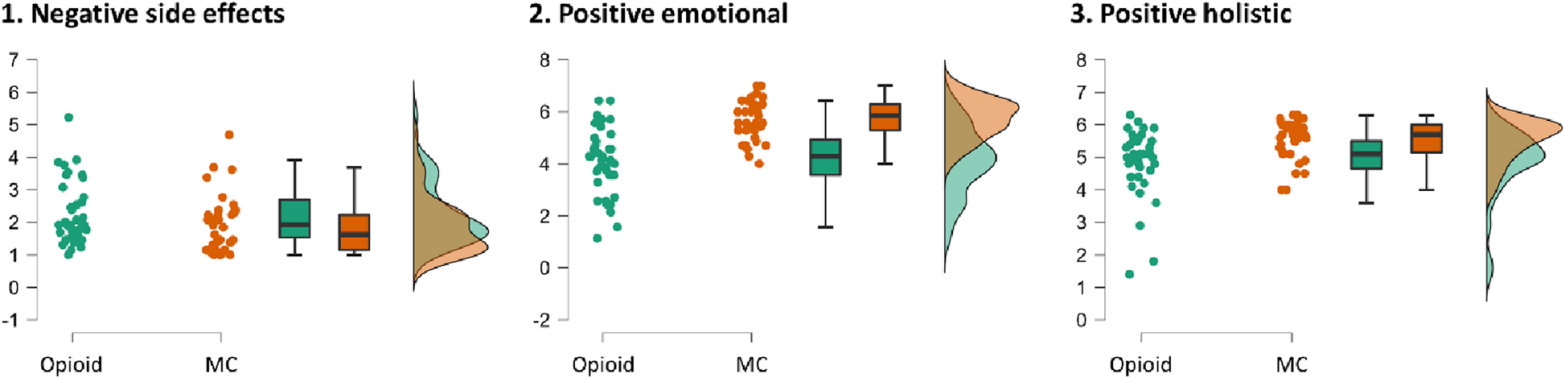
Raincloud plots of the group differences in the three factor scores, indicating most pronounced differences in Positive emotional effects, with higher ratings in the medical cannabis (MC) group. *Note*: The range in each factor is from 1 to 7, calculated as average to the respective experience questions which were rated on a 7-point Likert scale from “Completely of the opposite opinion” to “Completely of the same opinion”

The groups (*n* = 39 each) were compared regarding their perceived effects of the medicine (MC or opioids) using the averaged factor scores. In terms of Bonferroni-corrected p-value, the MC group showed higher scores on Positive Emotional and Holistic Effects, but no difference in Negative Effects. This was supported by the Bayes Factor, indicating decisive evidence (BF_10_ > 100) for more Positive Emotional Effects in the MC group, strong evidence (BF_10_ > 10) for more Positive Holistic Effects in the MC group, but only anecdotal evidence (BF_10_ < 3) of less Negative Side Effects in the MC group. The standardized effect size was large (0.71) for Positive Emotional and medium (0.47) for Positive Holistic. The results are summarized in Table 3 and Fig. 2. Regarding the perceived efficacy of MC and opioids in reducing pain intensity, the average rating to the intensity-related items 9 and 10 was 5.91 (SD = 0.87) in the MC group and 5.39 (SD = 1.31) in the opioid group (range from 1 to 7), with no group difference (W = 586.50, *p* = 0.078, BF_10_ = 1.19).

**Table 3:** Group differences in the three experience factors

	**MC**		**Opioid**						
	***M (SD)***	***CI***	***M (SD)***	***CI***	***W***	***p***	***p***_***B***_	***RBC***	**BF**_**10**_
1. Negative side effects	1.87 (0.87)	1.60—2.14	2.25 (.96)	1.95—2.55	964.00	0.042	0.13	0.27	1.89
2. Positive emotional	5.72 (0.75)	5.48—5.96	4.13 (1.28)	3.73—4.53	217.50	5.75E-08	< .001	-0.71	1466.00
3. Positive holistic	5.54 (0.62)	5.35—5.73	4.86 (1.04)	4.53—5.19	401.50	3.32E-04	< .001	-0.47	17.16

The range in each factor is from 1 to 7, calculated as average to the respective experience questions which were rated on a 7-point Likert scale from “Completely of the opposite opinion” to “Completely of the same opinion”

*MC* medical cannabis, *M* mean, *SD* standard deviation, *CI* = *95%* confidence interval, *RBC* Rank-Biserial Correlation; p_B_ = Bonferroni-corrected p-value (i.e., p-value multiplied by three)

As an additional group comparison, we examined if there is group difference in the average rating to the items 34 and 36 tapping on psychotic symptoms which were omitted from the factor analysis (see above). No group difference was observed (W = 657.00, BF_10_ = 0.39, *p* = 0.15).

**Fig. 3:**
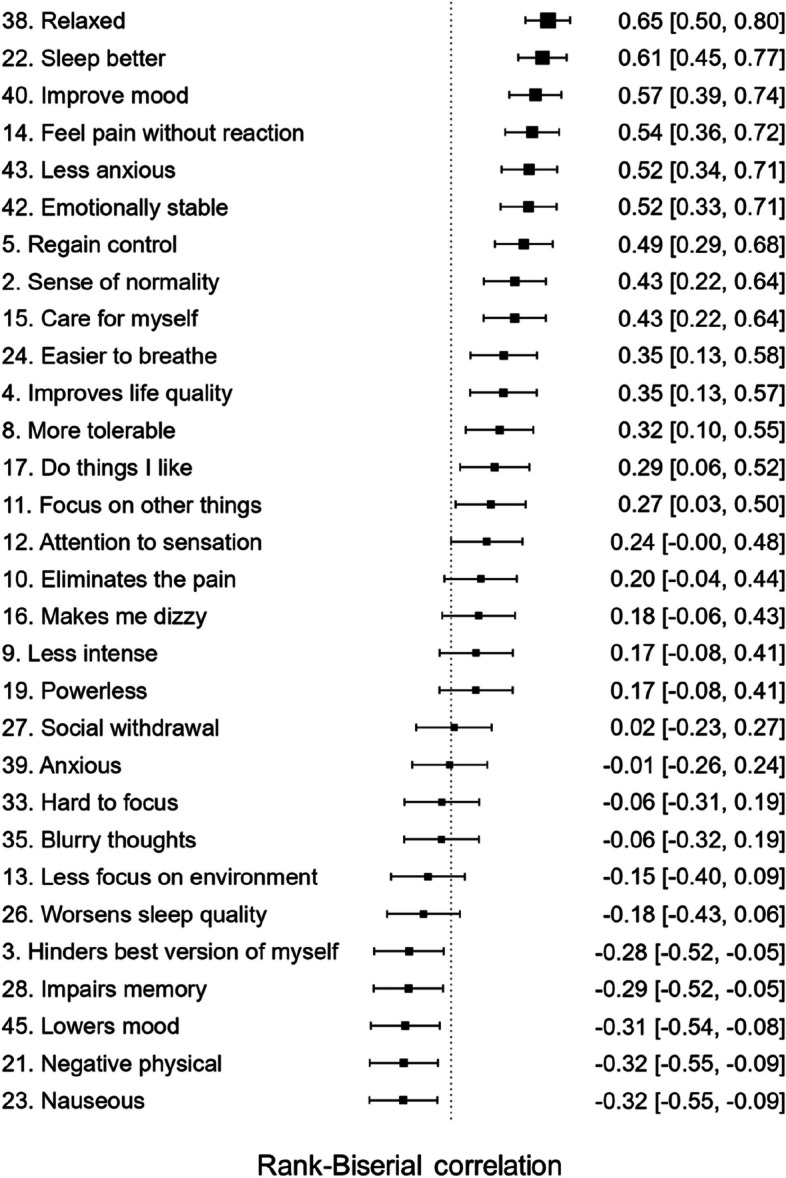
Explorative between-group comparisons of all the variables included in the final factor model, ordered by standardized effect size (Rank Biserial Correlation) from largest to smallest. *Note*: Larger effect size indicates higher rating in the medical cannabis group. Full item descriptions as well as group means and standard deviations for all the items can be found in Table 4. Items where the confidence interval does not overlap with the vertical line can be considered as significant

To see whether the group difference was larger for Positive Emotional Effects than for Positive Holistic Effects, we ran repeated measures ANOVA with the positive effect type (Emotional vs. Holistic) as the repeated factor, which showed an interaction with group and the factor type (F = 35.39, *p* = 7.74E-8, BF_incl_ > 100). That is, the group difference was larger for Positive Emotional Effects than for Positive Holistic Effects. Finally, to get a deeper insight into the experienced differences between the two medications, we conducted a non-planned analysis of the differences between the matched groups in ratings to all the questions from the final factor solution, summarized as a forest plot in Fig. 3. All items with means and standard deviations for the matched groups are summarized in Table 4.

**Table 4:** Ratings to all the experience statements with means and standard deviations from the matched groups (*n* = 39 per group)

**Item**	**Statement, starting with "The medicine…"**	**MC**		**Opioid**	
		*M*	*SD*	*M*	*SD*
1	provides me with a sense of relief	6.44	1.17	5.13	1.64
2	has enabled me to gain a sense of normality in my life	6.15	0.99	4.77	1.97
3	hinders me from being the best version of myself	1.85	1.31	2.95	2.15
4	has improved my life quality	6.59	0.79	5.97	1.20
5	has enabled me to regain a sense of control over my life	6.05	1.17	4.77	1.51
6	enables me to be the best version of myself	5.87	1.44	4.28	1.81
7	is optimal for me	6.64	0.71	5.77	1.44
8	makes the pain more tolerable	6.72	1.08	6.21	1.17
9	lessens the intensity of the pain	6.69	0.57	6.31	1.20
10	eliminates the pain	5.13	1.45	4.46	1.79
11	enables me to focus on other things beside the pain	6.46	0.82	5.74	1.53
12	enables me to pay more attention to sensations (like wind on my cheek, clock ticking, objects’s textures and patterns)^a^	5.28	1.41	4.51	1.81
13	makes it more difficult to focus on my environment and what happens around me	1.74	0.97	2.23	1.42
14	enables me to feel the pain without reacting to it	5.28	1.23	3.67	1.75
15	helps me to take care of myself	6.18	1.00	5.18	1.47
16	makes me dizzy	2.41	1.67	1.82	1.14
17	enables me to do the things I like	6.26	1.07	5.49	1.64
18	makes me drowsy	2.97	1.66	2.54	1.68
19	makes me feel powerless (lack energy)	2.08	1.18	1.87	1.47
20	enables me to enjoy the company of others	5.77	1.14	4.51	1.82
21	causes me negative physical symptoms	1.67	1.06	2.72	1.92
22	enables me to sleep better	6.15	1.09	4.13	2.02
23	makes me nauseous	1.28	0.65	1.77	0.93
24	makes it easier to breathe	4.90	1.33	3.82	1.55
25	makes me more active	5.77	1.35	4.85	1.93
26	worsens my sleep quality	1.64	1.18	2.36	1.81
27	makes me socially withdrawn	2.18	1.49	2.15	1.41
28	impairs my memory	2.56	1.35	3.41	1.79
29	makes it easier to maintain fous	5.74	1.21	4.69	1.42
30	makes my thoughts clearer	5.36	1.35	4.21	1.61
31	makes me feel intoxicated	2.59	1.70	1.62	0.99
32	makes me remember better	4.13	1.47	3.36	1.48
33	makes it hard to focus	2.10	1.25	2.36	1.51
34	causes hallucinations	1.44	0.91	1.18	0.56
35	makes my thoughts blurry	1.69	1.26	1.85	1.23
36	makes me paranoid	1.41	1.09	1.18	0.51
37	enables me to do complex tasks (e.g., work, cooking, etc.)	5.82	1.28	4.80	1.96
38	makes me more relaxed	6.33	1.16	4.59	1.70
39	makes me anxious	1.46	1.05	1.49	1.00
40	improves my mood	6.15	0.96	4.56	1.68
41	makes me believe more in the future	6.05	1.19	4.51	1.90
42	helps me feel more emotionally stable	5.39	1.39	3.90	1.54
43	makes me less anxious	5.85	1.39	4.26	1.79
44	produces euphoria	4.72	1.86	2.15	1.79
45	lowers my mood	1.59	1.07	2.28	1.34

Item 12 was adapted from the Five Facet Mindfulness Questionnaire

*MC* medical cannabis, *M* mean, *SD* standard deviation

It could be argued that recent opioid use in the MC group and recent MC use in the opioid group is a confounding factor in the analysis. Thus, a post hoc analysis was run to investigate whether the main group effects remain after excluding those in the groups who had used the other medication (i.e., MC in the opioid group or opioids in the MC group) during the last week. Two in the opioid group and 12 in the MC group were thus excluded, resulting in sample sizes of *n* = 37 and *n* = 27, respectively. In line with the main analysis, this comparison showed that the two groups did not differ in terms of negative side effects (W = 584, BF_10_ = 0.58, p_B_ = 0.77), but that the MC group showed higher ratings on the factors Positive Emotional (W = 135, BF_10_ = 542, p_B_ = 2.11E-06) and Positive Holistic (W = 281, BF_10_ = 12, p_B_ = 0.006).

## Discussion

Previous research indicates that MC may exert its therapeutic effect on pain through altering the pain experience holistically. That is, in addition to reducing pain intensity, MC may positively affect pain-related factors such as emotion and mood, functionality, and overall sense of well-being. By contrast, we hypothesized that the effect of opioids on pain is narrower, mainly targeting its intensity. We tested this hypothesis quantitatively in a survey study by assessing the perceived effects of MC or opioids in a group of Finnish chronic pain patients.

The main group comparison, based on the factor scores of the experience questions, indicated that MC and opioids did not differ in Negative Side Effects, but the effects of MC were rated as more positive in terms of the factors Positive Emotional and Positive Holistic Effects. Both MC and opioids were perceived to reduce pain intensity equally well. The group differences support the hypothesis that the effects of MC on pain are more holistic than those of opioids. The findings are in line with the previous qualitative research in support of the holistic positive effects of MC on pain (Chapkis 2007; Lavie-Ajayi & Shvartzman 2019). Moreover, the results corroborate previous data that MC alleviates pain-related negative emotion and increases pain tolerance (De Vita et al. 2018; Lee et al. 2013; Weizman et al. 2018), as well as findings that MC improves physical and social functionality and overall quality of life (Haroutounian et al. 2016; Pritchett et al. 2022).

The group differences were further examined by looking at the individual items exploratively (Fig. 3). The strongest group differences were, in order or magnitude, for relaxation, improved sleep, improved mood, and being able to feel pain without reacting to it. These items could reflect a broad range of cognitive-emotional processes. First, relaxation can be defined as relative absence of anxiety and physiological tension, manifested as calmness, peacefulness, and being at ease and has been associated with therapeutic benefits in pain management (Kwekkeboom & Gretarsdottir 2006). Second, pain is commonly associated with sleep disturbances, and sleep disturbance in turn can lead to worsening of pain and mood problems (Herrero Babiloni et al. 2020). Thus, sleep is a central node in the holistic network of pain-related experience factors. Third, mood disorders and pain frequently go together, and through acutely improving mood, MC could also decrease the unpleasantness of the pain sensation (cf. Tang et al. 2008). Fourth, being able to feel pain without reacting to it could be related to mindfulness processes such as detachment from the pain and pain acceptance, shown to be effective in pain management (Reiner, Tibi, & Lipsitz 2013).

It is noteworthy that the participants perceived MC and opioids as equally effective in reducing pain intensity. Given that opioids are known to be effective analgesics (Meske et al. 2018), this suggests that MC was perceived to have an analgesic effect, in addition to holistically altering the pain experience. This is in line with some previous reviews and meta analyses (Aviram & Samuelly-Leichtag 2017; Johal et al. 2020; Whiting et al. 2015), but inconsistent with others (Fisher et al. 2021; Gedin et al. 2022). These inconsistencies could stem from the wide range of cannabis-based medicines examined in previous research. It is possible that whole-plant cannabis flower, which was predominantly used by the patients in the present study, is more effective on pain intensity than isolated or synthetic cannabinoids. To illustrate, in the review of Fisher et al. (2021), only five of the 37 included studies examined cannabis flower, all with significant positive effects. Likewise, in Gedin et al. (2022) only six of the 20 studies that were included in the review dealt with whole plant-based products, all superior to placebo. By contrast, in both studies, the results concerning isolated and synthetic cannabinoids were mixed. Thus, the present results contribute to the cumulating evidence that whole-plant cannabis flower may be an effective analgesic.

In sum, the results lend support to the notion that the psychoactive effects of MC are relevant to its therapeutic effect on pain, in line with suggestions in previous literature (Chapkis 2007; Lavie-Ajayi & Shvartzman 2019; Lee et al. 2013; Stith et al. 2023). However, by “psychoactive” in this case we do not mean something that produces an altered state of consciousness in the sense of distorting one’s perception of reality and cognitive processes (Revonsuo et al. 2009), but instead something that holistically alters consciousness to a more positive direction, or towards “normality”. There were no indications that MC, despite its holistic effects on consciousness, was experienced to distort cognitive processes, but instead was perceived to improve memory, focus, and clarity of thought (see items 28–33 and 35, Table 4). This is in line with previous findings from a longitudinal study where MC use was associated with improved neurocognitive performance (Sagar et al. 2021). The results of the present study underline that the psychoactive effects of MC can be therapeutically positive and have beneficial effects on mood and functioning. However, this conclusion would require more robust testing, ideally in randomized controlled trials (RCT).

### Limitations

A central limitation of the present survey study pertains to the special status of MC in Finland. Although legal in principle, prescription for MC is very difficult to attain. Medical cannabis prescription rates have decreased in Finland from about 370 in 2017 to 240 in 2020 and to around 160 in 2021 (Harmaala 2022; Honkasalo 2022; Vihervaara & Hupli 2021). In the first half of 2022 there were less than 50 prescriptions (Harmaala 2022). For these reasons, we decided already in the preregistration to include in the study even those MC patients without prescription. This could increase the risk of recreational users participating in the survey. However, to control for this risk, we probed extensively about the patients’ medical background and the results indicated that the groups mainly did not differ in the severity and type of their pain condition.

Another risk related to the special status of MC in Finland is that, due to the negative perceptions about MC of medical doctors and authorities as well as severe difficulties in receiving a MC prescription, the patients may exaggerate the benefits of MC. However, systematic exaggeration of the benefits of MC would arguably also lead to underrating negative side effects, which was not the case here. Moreover, there was a differential effect in the two positive factors, supported by the ANOVA analysis, with the group difference being larger for the emotional factor than for the holistic factor. It is unlikely that this interaction effect would be due to systematic exaggeration of positive effects.

The small sample size is an obvious limitation of the present study and is mainly due to the very small MC population in Finland. This limits the generalizability and reliability of the results and motivates replication in a larger, independent sample in a country where the MC population is larger. However, it can be argued that the present sample is relatively representative of the larger population of chronic pain patients that use MC to manage the pain. For example, in a study on US chronic pain MC patients (*N* = 984) (Piper et al. 2017), the patients were 53% female, on average 49 years old, mainly had a vocational degree (38%), and mainly reported back/neck pain (91%), followed by neuropathic pain (30%). Most of them used MC as smoked (46%), followed by vaporized (23%). To compare, in our sample the participants were predominantly male (70%), of 43 years of age, mainly had vocational degree, and mainly suffered from neuropathic pain (60%) or other type of pain (60%) (note that we did not focus on pain location as Piper et al.). In our study, vaporizing was the most popular consumption method (40%), followed by smoking (30%). In sum, the present sample corresponds to some degree to the larger population of MC users, but there are also differences, motivating the use of a larger and more representative sample in future studies on this topic. Regarding the presence of “illegal” MC users in the present study, it is noteworthy that a Danish study (*N* = 3,021) focusing on medical use of cannabis (including pain) found that most of the participants (91%) had no prescription for the medicine, suggesting that this trend is common even in countries with legal medical cannabis (Kvamme et al. 2021).

Another limitation of the present study pertains to the experience questionnaire, which has not been previously validated. Although the present results indicate that it has good psychometric properties in terms of factor structure and reliability, the questionnaire should be tested for construct and convergent validity, which was not within the scope of the present paper.

In general, there are inherent limitations to survey studies, pertaining to factors such as insufficient control, non-experimental design, and possible confounding factors. On the other hand, survey studies on existing patient populations can also be considered as a more ecologically valid approach compared to RCTs (Schlag et al. 2022). Survey studies should not be seen as competing with RCTs, but instead the two approaches can be seen as mutually supportive. The present results highlight the importance of considering the holistic effects of MC on the pain experience also in RCT research.

## Conclusion

The results of the present study support the hypothesis that the effects of MC on pain experience are more holistic than those of opioids. MC may alleviate pain through affecting a broad range of pain-related experience experiental factors such as relaxation, improved sleep and mood, being able not to react to the pain, as well as a sense of control. These holistic effects of MC could explain the inconsistencies in clinical trials, where focus has mainly been on pain intensity instead of broader pain phenomenology. The results highlight the importance of taking these holistic effects into account in treating patients with MC, considering them as part of the therapeutic process.

### Supplementary Information

**Additional file 1.** 

**Additional file 2.** 

## Data Availability

The datasets generated and/or analyzed during the current study are available in the Open Science Framework repository, https://osf.io/8x5e6/.
