# Supplementary material for: The holistic effects of medical cannabis compared to opioids on pain experience in Finnish patients with chronic pain
Source: J Cannabis Res. 2023 Nov 9;5:38. doi: 10.1186/s42238-023-00207-7 (PMC10634036; doi:10.1186/s42238-023-00207-7)
Supplement: Supplementary file 2 — Additional file 2. [file 42238_2023_207_MOESM2_ESM.docx]

**<LANDING PAGE>**


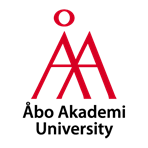


**Tutkimus lääkekannabiksen tai opiaattien/opioidien vaikutuksesta krooniseen kipuun**

Kärsitkö kroonisesta kivusta? Käytätkö sen hoitoon joko lääkekannabista tai opioideja/opiaatteja? Olet tervetullut osallistumaan tutkimukseemme koskien näiden lääkeaineiden vaikutusta kipukokemukseesi. Tutkimuksessa annat joitain taustatietoja itsestäsi sekä arvioit jommankumman lääkkeen (kannabis tai opioidi/opiaatti) vaikutuksia kipukokemukseesi. Vastaamiseen kuluu aikaa 30-45 minuuttia. Emme kerää mitään tunnistetietoja eikä sinua voida tunnistaa vastauksistasi. Tutkimuksen suorittaa tutkimusryhmä Åbo Akademilla ja vastaava tutkija on psykologi Jussi Jylkkä (PsT, FT). Voit osallistua tutkimukseen tämän linkin kautta: <https://abo.surveyanalytics.com/t/AJAw9ZOrDt>  Tutkimukseen voi osallistua 8. toukokuuta 2022 saakka. Reseptin puute ei ole este osallistumiselle.

Huomioithan, että kysely on suunnattu vain niille, jotka käyttävät kannabista tai opioideja ensisijaisesti kroonisen kivun hoitoon. Ethän siis vastaa kyselyyn, mikäli ensisijainen käyttötarkoituksesi on ns. viihdekäyttö tai muiden kuin kroonisesta kivusta johtuvien oireiden hallinta.

**En studie om effekten av medicinsk cannabis eller opiater/opioider på kronisk smärta**

Lider du av kronisk smärta? Använder du antingen medicinsk cannabis eller opioider/opiater för att behandla smärtan? Du är välkommen att delta i vår forskning om dessa läkemedels verkan på din smärtupplevelse. I forskningen ger du en del bakgrundsinformation om dig själv, samt utvärderar någotdera läkemedlets (cannabis eller opioid/opiat) verkan på din smärtupplevelse. Det tar 30-45 minuter att besvara frågeformuläret. Vi samlar inte in någon identifikationsinformation, och du kan inte igenkännas genom dina svar. Forskningen utförs av en forskningsgrupp vid Åbo Akademi och den ansvarande forskaren är Jussi Jylkkä (PsD, FD). Du kan delta i forskningen via denna länk: <https://abo.surveyanalytics.com/t/AJAw9ZOrDt> Deltagande är möjligt tills 8. Maj 2022. Du kan delta även om du inte har aktiv recept.

Vänligen notera att studie är riktad bara till människor som främst använder cannabis eller opiater/opioider för att behandla kronisk smärta. Vi ber dig att inte svara på enkäten om din användning är huvudsakligen nöjesanvändning eller behandling av andra symptom än smärta.

**Study on the effects of medical cannabis or opioids on chronic pain**

Do you suffer from chronic pain? Do you use either medical cannabis or opioids/opiates to treat it? You are welcome to participate in our study on the effect that these medications have on your experience of pain. In the study you will give some background information about yourself and estimate the effects of one of the medicines (medical cannabis or opioid/opiate) on your experience of pain. It takes 30-45 minutes to respond to the questionnaire. We do not collect any identification information about you, and you cannot be identified from your responses. The study is conducted by a research group at Åbo Akademi University, and the researcher-in-charge is Jussi Jylkkä (PsyD, PhD). You can participate in the study through this link: <https://abo.surveyanalytics.com/t/AJAw9ZOrDt> It is possible to participate until May 8^th^ 2022. You can participate even if you don’t have active prescription.

Please note that the study is intended only for those who use cannabis or opioids to treat chronic pain. Please do not answer the questionnaire if you primarily use cannabis recreationally or to manage other types of symptoms.

<COMPLETE QUESTIONNAIRE>

**Study on the subjectively felt effects of pain medication in persons who use medical cannabis or opiates/opioids**

This study is targeted to people suffering from chronic pain, and who use either medical cannabis or opiates/opioids for its treatment.

**Informed consent**

Thank you for your interest in our study! This is a study about the experienced effect of medical cannabis and opiates on chronic pain.  In our study, you will fill in a questionnaire and respond to questions regarding your use of medicine as well as your experience of its impact. You will also give demographic information such as gender, age, nationality, country of residence and details of your medicines and your anamnesis (medical history). Kindly observe that all information gathered for the study will be treated confidentially and we will ensure your anonymity; that is to say, it will not be possible to identify you through your answers. The IP address of those participating will not be stored. The responses are stored on a secure server and only the research group and the instructor have access to them with a password. Anonymous data may be made accessible for other researchers who might, in the future, be interested in analysing the data. If you feel uncomfortable with giving detailed information regarding your state of health, use of medicine and its effect on your experience of pain, it is not recommended that you participate in the study.

In order to participate in our study, you must be at least 18 years of age, live in Finland or have Finnish citizenship, and use either medical cannabis or opiates for chronic pain. Participation is voluntary. While you are responding, you can end your participation at any time by closing the questionnaire; you do not need to give a reason for ending your participation. The questionnaire takes approximately half an hour to fill in. Please reserve enough time to respond to the questions. You will not receive personal feedback after the study. You can fill in the questionnaire regardless of the type of your device, i.e., a computer, mobile phone, or tablet. Keep the device connected to the internet during the entire time that you are filling in the questionnaire. Please note that the survey will shut down automatically after 30 minutes if there is no screen activity.

This study is performed by students in psychology at Åbo Akademi University. If you have any questions regarding the study, you may send an e-mail to our instructor jussi.jylkka@abo.fi. All correspondence is treated confidentially. By clicking “OK”, you confirm that you have read and understood the above text, that you are at least 18 years of age, living in Finland or in possession of Finnish citizenship, and that you are willing to participate in the study.

**1. Background factors**

We want to emphasize that honest answers are critical and that the participant cannot be identified even in principle. We do not collect IP-addresses or other identifying information.

Citizenship

1. Finland
2. Other

Country of residency

1. Finland
2. Other

Age

|  |
| --- |

Sex

1. Male
2. Female
3. Other
4. Do not want to answer

Income level compared to average in country of residence (Finland)

1. substantially below average
2. slightly below average
3. average
4. slightly above average
5. substantially above average

What is the highest education level you have completed?

1. Grade school
2. Vocational school
3. Upper secondary school (high school)
4. Vocational university
5. Undergraduate degree
6. Graduate degree
7. Doctor
8. Other, specify __________

**Chronic Pain Questionnaire**

1. Have you experienced pain on most days in the past three (3) months?

1. Yes
2. No

2. Select the number that best describes your pain on average over the past week.

1. 0, no pain
2. 1
3. 2
4. 3
5. 4
6. 5
7. 6
8. 7
9. 8
10. 9
11. 10, worst possible pain

3. On the body map, please indicate all areas where you have had pain over the past week. <NOTE: THIS WAS PRESENTED AS A BODY MAP>

|  | Low | Medium | High |
| --- | --- | --- | --- |
| Head | ❏ | ❏ | ❏ |
| Nose | ❏ | ❏ | ❏ |
| Neck | ❏ | ❏ | ❏ |
| Right Shoulder | ❏ | ❏ | ❏ |
| Left shoulder | ❏ | ❏ | ❏ |
| Left chest | ❏ | ❏ | ❏ |
| Right Chest | ❏ | ❏ | ❏ |
| Left Bicep | ❏ | ❏ | ❏ |
| Right bicep | ❏ | ❏ | ❏ |
| Left forearm | ❏ | ❏ | ❏ |
| Right forearm | ❏ | ❏ | ❏ |
| Left hand | ❏ | ❏ | ❏ |
| Right hand | ❏ | ❏ | ❏ |
| Thorax | ❏ | ❏ | ❏ |
| Abdomen | ❏ | ❏ | ❏ |
| Groin | ❏ | ❏ | ❏ |
| Left Thigh | ❏ | ❏ | ❏ |
| Right Thigh | ❏ | ❏ | ❏ |
| Left Knee | ❏ | ❏ | ❏ |
| Right Knee | ❏ | ❏ | ❏ |
| Left Shin | ❏ | ❏ | ❏ |
| Right Shin | ❏ | ❏ | ❏ |
| Left foot | ❏ | ❏ | ❏ |
| Right Foot | ❏ | ❏ | ❏ |
| Head | ❏ | ❏ | ❏ |
| Neck | ❏ | ❏ | ❏ |
| Left Ear | ❏ | ❏ | ❏ |
| Right Ear | ❏ | ❏ | ❏ |
| Left Shoulder | ❏ | ❏ | ❏ |
| Right Shoulder | ❏ | ❏ | ❏ |
| Left Trapezius | ❏ | ❏ | ❏ |
| Right Trapezius | ❏ | ❏ | ❏ |
| Left Tricep | ❏ | ❏ | ❏ |
| Right Tricep | ❏ | ❏ | ❏ |
| Left elbow | ❏ | ❏ | ❏ |
| Right Elbow | ❏ | ❏ | ❏ |
| Left forearm | ❏ | ❏ | ❏ |
| Right forearm | ❏ | ❏ | ❏ |
| Right wrist | ❏ | ❏ | ❏ |
| Left wrist | ❏ | ❏ | ❏ |
| Center Back | ❏ | ❏ | ❏ |
| Lower Middle Back | ❏ | ❏ | ❏ |
| Lower Back | ❏ | ❏ | ❏ |
| Left Buttocks | ❏ | ❏ | ❏ |
| Right Buttocks | ❏ | ❏ | ❏ |
| Left Hamstring | ❏ | ❏ | ❏ |
| Right Hamstring | ❏ | ❏ | ❏ |
| Left Kneepit | ❏ | ❏ | ❏ |
| Right Kneepit | ❏ | ❏ | ❏ |
| Left Calf | ❏ | ❏ | ❏ |
| Right Calf | ❏ | ❏ | ❏ |
| Left heel | ❏ | ❏ | ❏ |
| Right heel | ❏ | ❏ | ❏ |

If the body map did not work, in the field below you may write in which part of your body you feel the pain.

|  |
| --- |

4. Did the pain feel like pins and needles?

1. Yes
2. No

5. Did the pain feel hot/burning?

1. Yes
2. No

6. Did the pain feel numb?

1. Yes
2. No

7. Did the pain feel like electrical shocks?

1. Yes
2. No

8. Is the pain made worse with the touch of clothing or bed sheets?

1. Yes
2. No

9. Is the pain limited to your joints?

1. Yes
2. No

Questions 10-12 ask about how much your pain may or may not have interfered with various aspects of your life in the past week (0 "not at all" - 10 "completely interferes"). Thinking about the past week, select the number that best describes how much your pain has interfered with your…

10. Usual activities (such as daily routine, walking, leisure or social activities, work, housework,

self-care)?

1. 0, not at all
2. 1
3. 2
4. 3
5. 4
6. 5
7. 6
8. 7
9. 8
10. 9
11. 10, completely interferes

11. Sleep?

1. 0, not at all
2. 1
3. 2
4. 3
5. 4
6. 5
7. 6
8. 7
9. 8
10. 9
11. 10, completely interferes

12. Mood?

1. 0, not at all
2. 1
3. 2
4. 3
5. 4
6. 5
7. 6
8. 7
9. 8
10. 9
11. 10, completely interferes

13. Did you have trouble thinking or remembering in the past week?

1. 0, no problem
2. 1
3. 2
4. 3
5. 4
6. 5
7. 6
8. 7
9. 8
10. 9
11. 10, severe problem

14. Were you sensitive to such things as bright lights, or loud noises, or smells in the past week?

1. 0, no problem
2. 1
3. 2
4. 3
5. 4
6. 5
7. 6
8. 7
9. 8
10. 9
11. 10, severe problem

**Mental wellbeing**

During the past two (2) weeks, how much (or how often) have you been bothered by the following problems? 0: none (not at all), 1: slight (rare, less than a day or two), 2: mild (several days), 3: moderate (more than half the days), 4: severe (nearly every day)

1. Little interest or pleasure in doing things?

1. 0, none
2. 1, slight
3. 2, mild
4. 3, moderate
5. 4, severe

2. Feeling down, depressed or hopeless?

1. 0, none
2. 1, slight
3. 2, mild
4. 3, moderate
5. 4, severe

3. Feeling more irritated, grouchy or angry than usual?

1. 0, none
2. 1, slight
3. 2, mild
4. 3, moderate
5. 4, severe

4. Sleeping less than usual, but still having a lot of energy?

1. 0, none
2. 1, slight
3. 2, mild
4. 3, moderate
5. 4, severe

5. Starting lots more projects than usual or doing more risky things than usual?

1. 0, none
2. 1, slight
3. 2, mild
4. 3, moderate
5. 4, severe

6. Feeling nervous, anxious, frightened, worried or on edge?

1. 0, none
2. 1, slight
3. 2, mild
4. 3, moderate
5. 4, severe

7. Feeling panic or being frightened?

1. 0, none
2. 1, slight
3. 2, mild
4. 3, moderate
5. 4, severe

8. Avoiding situations that make you anxious?

1. 0, none
2. 1, slight
3. 2, mild
4. 3, moderate
5. 4, severe

9. Unexplained aches and pains (e.g. head, back, joints, abdomen, legs)?

1. 0, none
2. 1, slight
3. 2, mild
4. 3, moderate
5. 4, severe

10. Feeling that your illnesses are not being taken seriously enough?

1. 0, none
2. 1, slight
3. 2, mild
4. 3, moderate
5. 4, severe

11. Thoughts of actually hurting yourself?

1. 0, none
2. 1, slight
3. 2, mild
4. 3, moderate
5. 4, severe

12. Hearing things other people couldn’t hear, such as voices even when no one was around?

1. 0, none
2. 1, slight
3. 2, mild
4. 3, moderate
5. 4, severe

13. Feeling that someone could hear your thoughts, or that you could hear what another person was thinking?

1. 0, none
2. 1, slight
3. 2, mild
4. 3, moderate
5. 4, severe

14. Problems with sleep that affected your sleep quality overall?

1. 0, none
2. 1, slight
3. 2, mild
4. 3, moderate
5. 4, severe

15. Problems with memory (e.g. learning new information) or with location (e.g. finding your way home)?

1. 0, none
2. 1, slight
3. 2, mild
4. 3, moderate
5. 4, severe

16. Unpleasant thoughts, urges, or images that repeatedly enter your mind?

1. 0, none
2. 1, slight
3. 2, mild
4. 3, moderate
5. 4, severe

17. Feeling driven to perform certain behaviors or mental acts over and over again?

1. 0, none
2. 1, slight
3. 2, mild
4. 3, moderate
5. 4, severe

18. Feeling detached or distant from yourself, your body, your physical surroundings, or your memories?

1. 0, none
2. 1, slight
3. 2, mild
4. 3, moderate
5. 4, severe

19. Not knowing who you really are or what you want out of life?

1. 0, none
2. 1, slight
3. 2, mild
4. 3, moderate
5. 4, severe

20. Not feeling close to other people or enjoying your relationships with them?

1. 0, none
2. 1, slight
3. 2, mild
4. 3, moderate
5. 4, severe

21. Drinking at least 4 drinks of any kind of alcohol in a single day?

1. 0, none
2. 1, slight
3. 2, mild
4. 3, moderate
5. 4, severe

22. Smoking any cigarettes, a cigar, or pipe, or using snuff or chewing tobacco?

1. 0, none
2. 1, slight
3. 2, mild
4. 3, moderate
5. 4, severe

23. Using any of the following medicines on your own, that is, without a doctor’s prescription, in greater amounts or longer than prescribed, e.g. painkillers (like Vicodin), stimulants (like Ritalin or Adderall), sedatives or tranquilizers (like sleeping pills or Valium), or drugs like marijuana, cocaine or crack?

1. 0, none
2. 1, slight
3. 2, mild
4. 3, moderate
5. 4, severe

**2. Background related to pain**

Please specify below, what kind of pain you suffer from.

1. nociceptive pain (other than cancer-related), (tissue injury, e.g. musculoskeletal pain, arthrosis-related pain, ischemic or inflammation pain)
2. neuropathic (e.g. diabetic, or other polyneuropathy, injury, impingement, central (such as multiple sclerosis, post-stroke or spinal cord injury))
3. idiopathic pain (pain with unknown cause, e.g. fibromyalgia)
4. chronic pain disorder
5. cancer-related pain
6. visceral pain, e.g. irritable bowel syndrome, chronic pancreatitis, dysmenorrhea, inflammatory bowel disease
7. Other, what __________

Do you have a medical doctor’s diagnosis for the pain condition?

1. Yes
2. No

Please write the ICD-10 code for your diagnosis, if you know it:

|  |
| --- |

How long have you experienced chronic pain? Please give such an accurate month- or yearly based estimation of the duration of your pain as possible. Please remember to specify in your answer, whether your answers are given in months or years (months = mon ; years = yrs).

|  |
| --- |

**3. Treatment of pain**

Do you use opiates/opioids for your pain? <NOTE: DETAILED QUESTIONS ABOUT OPIATE USE BELOW ARE SHOWN ONLY WHEN THE ANSWER HERE IS “YES”>

1. Yes
2. No

What is the type of your opiate/opioid?

1. Weak (e.g. codeine, tramadol)
2. Medium (e.g. burprenorphine)
3. Strong (e.g. fentanyl, hydromorphone, metadone, morphine, oxycodone)

How long have you used this type of medication? Please specify whether you mean months or years.

|  |
| --- |

Have you used this medication within the last week?

1. Yes
2. No

How many times a week have you used this medication?

|  |
| --- |

How many mg per dose?

|  |
| --- |

Do you have a prescription for the medication?

1. Yes
2. Never had
3. Had, but it was discontinued

Do you use medical cannabis for your pain? <NOTE: DETAILED QUESTIONS ABOUT MEDICAL CANNABIS USE BELOW ARE SHOWN ONLY WHEN THE ANSWER HERE IS “YES”>

1. Yes
2. No

What is the type of your medical cannabis?

1. Bedrocan
2. Bediol
3. Sativex
4. Don’t know
5. Other, please specify (e.g. what variety) __________

If possible, please evaluate the THC:CBD ratio in the strain you use .

1. Balanced (50/50)
2. THC dominant
3. CBD dominant
4. Don’t know

How long have you used this medication? Please specify whether you mean months or years.

|  |
| --- |

Have you used this medication within the last week?

1. Yes
2. No

How many times a week have you used this medication?

|  |
| --- |

Please tell how much of the preparate you use per dose, e.g. “1 flower head” or “6 sprays of Sativex”.

|  |
| --- |

In which form do you use the medication?

1. Eaten
2. Vaporized
3. Smoked
4. Other, what?

Do you have a prescription for the medication?

1. Yes
2. Never had
3. Had, but was discontinued

If it was discontinued, what year was this?

|  |
| --- |

During the past week, have you used some other medication for your pain? Please select from below all options which apply to you.

1. Antidepressants (tricyclic and SNRI e.g. Triptyl, Doxal, Surmontil, Anafranil)
2. Nonsteroidal anti-inflammatory medications (NSAIDs) (e.g. Burana, Pronaxen, Celecoxib, Mobic, Arcoxia)
3. Paracetamol (e.g. Panadol)
4. Anti-epileptic medication (gabapentinoids (pregabalin and gabapentin), carbamazepine, oxcarbazepine)
5. Have not used
6. Other (e.g. capsaicin plaster, superficial or injected anesthesia, botulinum injections, ketamine-infusion treatments), specify. __________

Do you receive non-medical treatment for your pain? Please select from below all options which apply to you.

1. Psychotherapy
2. Physiotherapy
3. Mindfulness
4. I do not receive non-medical tretment
5. Other, specify. __________

|  |
| --- |

Select below all medications/drugs which you have used, in the past year, for a reason other than medical use.

1. Amphetamine
2. Metamphetamine
3. Cocaine
4. Cannabis
5. Psychedelics (LSD, psilocybin, mescaline)
6. Opioids (e.g. buprenorphine, heroin, fentanyl)
7. MDMA
8. Design drugs
9. Solvents
10. Have not used
11. Other, what? __________

**4. Questionnaire on pain experience**

We want to emphasize that honest answers are critical and that the participant cannot be identified even in principle. We do not collect IP-addresses or other identifying information.  We are interested in how you experience that the medication you use affects the pain. Please choose below which medication (medical cannabis or opioids/opiates) your answers concern. Please only judge the effects of the medicine you choose.

Select which medicine your responses are about.

1. Medical cannabis
2. Opioids or opiates
3. Other, what: __________

IMPORTANT! The medicine you select here is referred to as “The medicine” below.

1 The medicine provides me with a sense of relief

|  | Completely of the opposite opinion | Mostly of the opposite opinion | Somewhat of the opposite opinion | Neither of the opposite nor the same opinion | Somewhat of the same opinion | Mostly of the same opinion | Completely of the same opinion |
| --- | --- | --- | --- | --- | --- | --- | --- |
|  | ❏ | ❏ | ❏ | ❏ | ❏ | ❏ | ❏ |

2 The medicine has enabled me to gain a sense of normality in my life

|  | Completely of the opposite opinion | Mostly of the opposite opinion | Somewhat of the opposite opinion | Neither of the opposite nor the same opinion | Somewhat of the same opinion | Mostly of the same opinion | Completely of the same opinion |
| --- | --- | --- | --- | --- | --- | --- | --- |
|  | ❏ | ❏ | ❏ | ❏ | ❏ | ❏ | ❏ |

3 The medicine hinders me from being the best version of myself

|  | Completely of the opposite opinion | Mostly of the opposite opinion | Somewhat of the opposite opinion | Neither of the opposite nor the same opinion | Somewhat of the same opinion | Mostly of the same opinion | Completely of the same opinion |
| --- | --- | --- | --- | --- | --- | --- | --- |
|  | ❏ | ❏ | ❏ | ❏ | ❏ | ❏ | ❏ |

4 The medicine has improved my life quality

|  | Completely of the opposite opinion | Mostly of the opposite opinion | Somewhat of the opposite opinion | Neither of the opposite nor the same opinion | Somewhat of the same opinion | Mostly of the same opinion | Completely of the same opinion |
| --- | --- | --- | --- | --- | --- | --- | --- |
|  | ❏ | ❏ | ❏ | ❏ | ❏ | ❏ | ❏ |

5 The medicine has enabled me to regain a sense of control over my life

|  | Completely of the opposite opinion | Mostly of the opposite opinion | Somewhat of the opposite opinion | Neither of the opposite nor the same opinion | Somewhat of the same opinion | Mostly of the same opinion | Completely of the same opinion |
| --- | --- | --- | --- | --- | --- | --- | --- |
|  | ❏ | ❏ | ❏ | ❏ | ❏ | ❏ | ❏ |

6 The medicine enables me to be the best version of myself

|  | Completely of the opposite opinion | Mostly of the opposite opinion | Somewhat of the opposite opinion | Neither of the opposite nor the same opinion | Somewhat of the same opinion | Mostly of the same opinion | Completely of the same opinion |
| --- | --- | --- | --- | --- | --- | --- | --- |
|  | ❏ | ❏ | ❏ | ❏ | ❏ | ❏ | ❏ |

7 The medicine is optimal for me

|  | Completely of the opposite opinion | Mostly of the opposite opinion | Somewhat of the opposite opinion | Neither of the opposite nor the same opinion | Somewhat of the same opinion | Mostly of the same opinion | Completely of the same opinion |
| --- | --- | --- | --- | --- | --- | --- | --- |
|  | ❏ | ❏ | ❏ | ❏ | ❏ | ❏ | ❏ |

8 The medicine makes the pain more tolerable

|  | Completely of the opposite opinion | Mostly of the opposite opinion | Somewhat of the opposite opinion | Neither of the opposite nor the same opinion | Somewhat of the same opinion | Mostly of the same opinion | Completely of the same opinion |
| --- | --- | --- | --- | --- | --- | --- | --- |
|  | ❏ | ❏ | ❏ | ❏ | ❏ | ❏ | ❏ |

9 The medicine lessens the intensity of the pain

|  | Completely of the opposite opinion | Mostly of the opposite opinion | Somewhat of the opposite opinion | Neither of the opposite nor the same opinion | Somewhat of the same opinion | Mostly of the same opinion | Completely of the same opinion |
| --- | --- | --- | --- | --- | --- | --- | --- |
|  | ❏ | ❏ | ❏ | ❏ | ❏ | ❏ | ❏ |

10 The medicine eliminates the pain

|  | Completely of the opposite opinion | Mostly of the opposite opinion | Somewhat of the opposite opinion | Neither of the opposite nor the same opinion | Somewhat of the same opinion | Mostly of the same opinion | Completely of the same opinion |
| --- | --- | --- | --- | --- | --- | --- | --- |
|  | ❏ | ❏ | ❏ | ❏ | ❏ | ❏ | ❏ |

11 The medicine enables me to focus on other things beside the pain

|  | Completely of the opposite opinion | Mostly of the opposite opinion | Somewhat of the opposite opinion | Neither of the opposite nor the same opinion | Somewhat of the same opinion | Mostly of the same opinion | Completely of the same opinion |
| --- | --- | --- | --- | --- | --- | --- | --- |
|  | ❏ | ❏ | ❏ | ❏ | ❏ | ❏ | ❏ |

12 The medicine enables me to pay more attention to sensations (like wind on my cheek, clock ticking, objects’s textures and patterns)

|  | Completely of the opposite opinion | Mostly of the opposite opinion | Somewhat of the opposite opinion | Neither of the opposite nor the same opinion | Somewhat of the same opinion | Mostly of the same opinion | Completely of the same opinion |
| --- | --- | --- | --- | --- | --- | --- | --- |
|  | ❏ | ❏ | ❏ | ❏ | ❏ | ❏ | ❏ |

13 The medicine makes it more difficult to focus on my environment and what happens around me

|  | Completely of the opposite opinion | Mostly of the opposite opinion | Somewhat of the opposite opinion | Neither of the opposite nor the same opinion | Somewhat of the same opinion | Mostly of the same opinion | Completely of the same opinion |
| --- | --- | --- | --- | --- | --- | --- | --- |
|  | ❏ | ❏ | ❏ | ❏ | ❏ | ❏ | ❏ |

14 The medicine enables me to feel the pain without reacting to it

|  | Completely of the opposite opinion | Mostly of the opposite opinion | Somewhat of the opposite opinion | Neither of the opposite nor the same opinion | Somewhat of the same opinion | Mostly of the same opinion | Completely of the same opinion |
| --- | --- | --- | --- | --- | --- | --- | --- |
|  | ❏ | ❏ | ❏ | ❏ | ❏ | ❏ | ❏ |

15 The medicine helps me to take care of myself

|  | Completely of the opposite opinion | Mostly of the opposite opinion | Somewhat of the opposite opinion | Neither of the opposite nor the same opinion | Somewhat of the same opinion | Mostly of the same opinion | Completely of the same opinion |
| --- | --- | --- | --- | --- | --- | --- | --- |
|  | ❏ | ❏ | ❏ | ❏ | ❏ | ❏ | ❏ |

16 The medicine makes me dizzy

|  | Completely of the opposite opinion | Mostly of the opposite opinion | Somewhat of the opposite opinion | Neither of the opposite nor the same opinion | Somewhat of the same opinion | Mostly of the same opinion | Completely of the same opinion |
| --- | --- | --- | --- | --- | --- | --- | --- |
|  | ❏ | ❏ | ❏ | ❏ | ❏ | ❏ | ❏ |

17 The medicine enables me to do the things I like

|  | Completely of the opposite opinion | Mostly of the opposite opinion | Somewhat of the opposite opinion | Neither of the opposite nor the same opinion | Somewhat of the same opinion | Mostly of the same opinion | Completely of the same opinion |
| --- | --- | --- | --- | --- | --- | --- | --- |
|  | ❏ | ❏ | ❏ | ❏ | ❏ | ❏ | ❏ |

18 The medicine makes me drowsy

|  | Completely of the opposite opinion | Mostly of the opposite opinion | Somewhat of the opposite opinion | Neither of the opposite nor the same opinion | Somewhat of the same opinion | Mostly of the same opinion | Completely of the same opinion |
| --- | --- | --- | --- | --- | --- | --- | --- |
|  | ❏ | ❏ | ❏ | ❏ | ❏ | ❏ | ❏ |

19 The medicine makes me feel powerless (lack energy)

|  | Completely of the opposite opinion | Mostly of the opposite opinion | Somewhat of the opposite opinion | Neither of the opposite nor the same opinion | Somewhat of the same opinion | Mostly of the same opinion | Completely of the same opinion |
| --- | --- | --- | --- | --- | --- | --- | --- |
|  | ❏ | ❏ | ❏ | ❏ | ❏ | ❏ | ❏ |

20 The medicine enables me to enjoy the company of others

|  | Completely of the opposite opinion | Mostly of the opposite opinion | Somewhat of the opposite opinion | Neither of the opposite nor the same opinion | Somewhat of the same opinion | Mostly of the same opinion | Completely of the same opinion |
| --- | --- | --- | --- | --- | --- | --- | --- |
|  | ❏ | ❏ | ❏ | ❏ | ❏ | ❏ | ❏ |

21 The medicine causes me negative physical symptoms

|  | Completely of the opposite opinion | Mostly of the opposite opinion | Somewhat of the opposite opinion | Neither of the opposite nor the same opinion | Somewhat of the same opinion | Mostly of the same opinion | Completely of the same opinion |
| --- | --- | --- | --- | --- | --- | --- | --- |
|  | ❏ | ❏ | ❏ | ❏ | ❏ | ❏ | ❏ |

22 The medicine enables me to sleep better

|  | Completely of the opposite opinion | Mostly of the opposite opinion | Somewhat of the opposite opinion | Neither of the opposite nor the same opinion | Somewhat of the same opinion | Mosly of the same opinion | Completely of the same opinion |
| --- | --- | --- | --- | --- | --- | --- | --- |
|  | ❏ | ❏ | ❏ | ❏ | ❏ | ❏ | ❏ |

23 The medicine makes me nauseous

|  | Completely of the opposite opinion | Mostly of the opposite opinion | Somewhat of the opposite opinion | Neither of the opposite nor the same opinion | Somewhat of the same opinion | Mostly of the same opinion | Completely of the same opinion |
| --- | --- | --- | --- | --- | --- | --- | --- |
|  | ❏ | ❏ | ❏ | ❏ | ❏ | ❏ | ❏ |

24 The medicine makes it easier to breathe

|  | Completely of the opposite opinion | Mostly of the opposite opinion | Somewhat of the opposite opinion | Neither of the opposite nor the same opinion | Somewhat of the same opinion | Mostly of the same opinion | Completely of the same opinion |
| --- | --- | --- | --- | --- | --- | --- | --- |
|  | ❏ | ❏ | ❏ | ❏ | ❏ | ❏ | ❏ |

25 The medicine makes me more active

|  | Completely of the opposite opinion | Mostly of the opposite opinion | Somewhat of the opposite opinion | Neither of the opposite nor the same opinion | Somewhat of the same opinion | Mostly of the same opinion | Completely of the same opinion |
| --- | --- | --- | --- | --- | --- | --- | --- |
|  | ❏ | ❏ | ❏ | ❏ | ❏ | ❏ | ❏ |

26 The medicine worsens my sleep quality

|  | Completely of the opposite opinion | Mostly of the opposite opinion | Somewhat of the opposite opinion | Neither of the opposite nor the same opinion | Somewhat of the same opinion | Mostly of the same opinion | Completely of the same opinion |
| --- | --- | --- | --- | --- | --- | --- | --- |
|  | ❏ | ❏ | ❏ | ❏ | ❏ | ❏ | ❏ |

27 The medicine makes me socially withdrawn

|  | Completely of the opposite opinion | Mostly of the opposite opinion | Somewhat of the opposite opinion | Neither of the opposite nor the same opinion | Somewhat of the same opinion | Mostly of the same opinion | Completely of the same opinion |
| --- | --- | --- | --- | --- | --- | --- | --- |
|  | ❏ | ❏ | ❏ | ❏ | ❏ | ❏ | ❏ |

28 The medicine impairs my memory

|  | Completely of the opposite opinion | Mostly of the opposite opinion | Somewhat of the opposite opinion | Neither of the opposite nor the same opinion | Somewhat of the same opinion | Mostly of the same opinion | Completely of the same opinion |
| --- | --- | --- | --- | --- | --- | --- | --- |
|  | ❏ | ❏ | ❏ | ❏ | ❏ | ❏ | ❏ |

29 The medicine makes it easier to maintain fous

|  | Completely of the opposite opinion | Mostly of the opposite opinion | Somewhat of the opposite opinion | Neither of the opposite nor the same opinion | Somewhat of the same opinion | Mostly of the same opinion | Completely of the same opinion |
| --- | --- | --- | --- | --- | --- | --- | --- |
|  | ❏ | ❏ | ❏ | ❏ | ❏ | ❏ | ❏ |

30 The medicine makes my thoughts clearer

|  | Completely of the opposite opinion | Mostly of the opposite opinion | Somewhat of the opposite opinion | Neither of the opposite nor the same opinion | Somewhat of the same opinion | Mostly of the same opinion | Completely of the same opinion |
| --- | --- | --- | --- | --- | --- | --- | --- |
|  | ❏ | ❏ | ❏ | ❏ | ❏ | ❏ | ❏ |

31 The medicine makes me feel intoxicated

|  | Completely of the opposite opinion | Mostly of the opposite opinion | Somewhat of the opposite opinion | Neither of the opposite nor the same opinion | Somewhat of the same opinion | Mostly of the same opinion | Completely of the same opinion |
| --- | --- | --- | --- | --- | --- | --- | --- |
|  | ❏ | ❏ | ❏ | ❏ | ❏ | ❏ | ❏ |

32 The medicine makes me remember better

|  | Completely of the opposite opinion | Mostly of the opposite opinion | Somewhat of the opposite opinion | Neither of the opposite nor the same opinion | Somewhat of the same opinion | Mostly of the same opinion | Completely of the same opinion |
| --- | --- | --- | --- | --- | --- | --- | --- |
|  | ❏ | ❏ | ❏ | ❏ | ❏ | ❏ | ❏ |

33 The medicine makes it hard to focus

|  | Completely of the opposite opinion | Mostly of the opposite opinion | Somewhat of the opposite opinion | Neither of the opposite nor the same opinion | Somewhat of the same opinion | Mostly of the same opinion | Completely of the same opinion |
| --- | --- | --- | --- | --- | --- | --- | --- |
|  | ❏ | ❏ | ❏ | ❏ | ❏ | ❏ | ❏ |

34 The medicine causes hallucinations

|  | Completely of the opposite opinion | Mostly of the opposite opinion | Somewhat of the opposite opinion | Neither of the opposite nor the same opinion | Somewhat of the same opinion | Mostly of the same opinion | Completely of the same opinion |
| --- | --- | --- | --- | --- | --- | --- | --- |
|  | ❏ | ❏ | ❏ | ❏ | ❏ | ❏ | ❏ |

35 The medicine makes my thoughts blurry

|  | Completely of the opposite opinion | Mostly of the opposite opinion | Somewhat of the opposite opinion | Neither of the opposite nor the same opinion | Somewhat of the same opinion | Mostly of the same opinion | Completely of the same opinion |
| --- | --- | --- | --- | --- | --- | --- | --- |
|  | ❏ | ❏ | ❏ | ❏ | ❏ | ❏ | ❏ |

36 The medicine makes me paranoid

|  | Completely of the opposite opinion | Mostly of the opposite opinion | Somewhat of the opposite opinion | Neither of the opposite nor the same opinion | Somewhat of the same opinion | Mostly of the same opinion | Completely of the same opinion |
| --- | --- | --- | --- | --- | --- | --- | --- |
|  | ❏ | ❏ | ❏ | ❏ | ❏ | ❏ | ❏ |

37 The medicine enables me to do complex tasks (e.g., work, cooking, etc.)

|  | Completely of the opposite opinion | Mostly of the opposite opinion | Somewhat of the opposite opinion | Neither of the opposite nor the same opinion | Somewhat of the same opinion | Mostly of the same opinion | Completely of the same opinion |
| --- | --- | --- | --- | --- | --- | --- | --- |
|  | ❏ | ❏ | ❏ | ❏ | ❏ | ❏ | ❏ |

38 The medicine makes me more relaxed

|  | Completely of the opposite opinion | Mostly of the opposite opinion | Somewhat of the opposite opinion | Neither of the opposite nor the same opinion | Somewhat of the same opinion | Mostly of the same opinion | Completely of the same opinion |
| --- | --- | --- | --- | --- | --- | --- | --- |
|  | ❏ | ❏ | ❏ | ❏ | ❏ | ❏ | ❏ |

39 The medicine makes me anxious

|  | Completely of the opposite opinion | Mostly of the opposite opinion | Somewhat of the opposite opinion | Neither of the opposite nor the same opinion | Somewhat of the same opinion | Mostly of the same opinion | Completely of the same opinion |
| --- | --- | --- | --- | --- | --- | --- | --- |
|  | ❏ | ❏ | ❏ | ❏ | ❏ | ❏ | ❏ |

40  The medicine improves my mood

|  | Completely of the opposite opinion | Mostly of the opposite opinion | Somewhat of the opposite opinion | Neither of the opposite nor the same opinion | Somewhat of the same opinion | Mostly of the same opinion | Completely of the same opinion |
| --- | --- | --- | --- | --- | --- | --- | --- |
|  | ❏ | ❏ | ❏ | ❏ | ❏ | ❏ | ❏ |

41 The medicine makes me believe more in the future

|  | Completely of the opposite opinion | Mostly of the opposite opinion | Somewhat of the opposite opinion | Neither of the opposite nor the same opinion | Somewhat of the same opinion | Mostly of the same opinion | Completely of the same opinion |
| --- | --- | --- | --- | --- | --- | --- | --- |
|  | ❏ | ❏ | ❏ | ❏ | ❏ | ❏ | ❏ |

42 The medicine helps me feel more emotionally stable

|  | Completely of the opposite opinion | Mostly of the opposite opinion | Somewhat of the opposite opinion | Neither of the opposite nor the same opinion | Somewhat of the same opinion | Mostly of the same opinion | Completely of the same opinion |
| --- | --- | --- | --- | --- | --- | --- | --- |
|  | ❏ | ❏ | ❏ | ❏ | ❏ | ❏ | ❏ |

43 The medicine makes me less anxious

|  | Completely of the opposite opinion | Mostly of the opposite opinion | Somewhat of the opposite opinion | Neither of the opposite nor the same opinion | Somewhat of the same opinion | Mostly of the same opinion | Completely of the same opinion |
| --- | --- | --- | --- | --- | --- | --- | --- |
|  | ❏ | ❏ | ❏ | ❏ | ❏ | ❏ | ❏ |

44 The medicine produces euphoria

|  | Completely of the opposite opinion | Mostly of the opposite opinion | Somewhat of the opposite opinion | Neither of the opposite nor the same opinion | Somewhat of the same opinion | Mostly of the same opinion | Completely of the same opinion |
| --- | --- | --- | --- | --- | --- | --- | --- |
|  | ❏ | ❏ | ❏ | ❏ | ❏ | ❏ | ❏ |

45 The medicine lowers my mood

|  | Completely of the opposite opinion | Mostly of the opposite opinion | Somewhat of the opposite opinion | Neither of the opposite nor the same opinion | Somewhat of the same opinion | Mostly of the same opinion | Completely of the same opinion |
| --- | --- | --- | --- | --- | --- | --- | --- |
|  | ❏ | ❏ | ❏ | ❏ | ❏ | ❏ | ❏ |

**5. Severity of Dependence Scale (SDS)**

Choose the answer that best applies to how you have felt about your use of the medicine over the last twelve months. Note, that we here refer to the medicine (medical cannabis or opiates/opioids) that you chose before and whose effects you assessed.

Do you think your use of the medication was out of control?

|  | Never or almost never | Sometimes | Often | Nearly always or always |
| --- | --- | --- | --- | --- |
|  | ❏ | ❏ | ❏ | ❏ |

Does the prospect of missing a dose make you feel anxious or worried?

|  | Never or almost never | Sometimes | Often | Nearly always or always |
| --- | --- | --- | --- | --- |
|  | ❏ | ❏ | ❏ | ❏ |

Do you worry about your use of the medication?

|  | Never or almost never | Sometimes | Often | Nearly always or always |
| --- | --- | --- | --- | --- |
|  | ❏ | ❏ | ❏ | ❏ |

Do you wish you could stop?

|  | Never or almost never | Sometimes | Often | Nearly always or always |
| --- | --- | --- | --- | --- |
|  | ❏ | ❏ | ❏ | ❏ |

How difficult did you find it to stop or go without the medication?

|  | Not difficult | Quite difficult | Very difficult | Impossible |
| --- | --- | --- | --- | --- |
|  | ❏ | ❏ | ❏ | ❏ |

If you wish, you can comment on the Severity of Dependence scale or your answers in the field below.

|  |
| --- |

**6. Change in comparison with previous treatment** <NOTE: THIS DATA WAS NOT ANALYZED IN THE STUDY “The holistic effects of medical cannabis compared to opioids on pain experience in Finnish patients with chronic pain”>

You can answer this section if you have had another medication or treatment for your pain before your current medicine. We are interested in how you experience your current medicine in comparison to the previous one. Answering this section is optional. If you do not wish to answer, scroll downwards and continue until you reach the "Finished" button, click on it and send your answers to us.

Have you had another medication or treatment for your pain before your current one?

1. Yes
2. No

What medication or treatment have you had for your pain before your current medicine?

1. Opiates/opioids
2. Nonsteroidal anti-inflammatory medications
3. Glucocorticoids (cortisone preparations)
4. Medication affecting the central nervous system
5. Mindfulness Therapy
6. Acceptance and Commitment Therapy
7. No earlier treatment
8. Other, what: __________

If you have had many previous medications or treatments, please specify below with which treatment you will compare your current medicine.

|  |
| --- |

On a scale of 1-7, indicate how the following statements reflect your situation.

My functioning is better with the current medicine than with the previous one.

|  | Completely of the opposite opinion | Mostly of the opposite opinion | Somewhat of the opposite opinion | Neither of the opposite nor the same opinion | Somewhat of the same opinion | Mostly of the same opinion | Completely of the same opinion |
| --- | --- | --- | --- | --- | --- | --- | --- |
|  | ❏ | ❏ | ❏ | ❏ | ❏ | ❏ | ❏ |

I sleep better with my current medicine than with the previous one.

|  | Completely of the opposite opinion | Mostly of the opposite opinion | Somewhat of the opposite opinion | Neither of the opposite nor the same opinion | Somewhat of the same opinion | Mostly of the same opinion | Completely of the same opinion |
| --- | --- | --- | --- | --- | --- | --- | --- |
|  | ❏ | ❏ | ❏ | ❏ | ❏ | ❏ | ❏ |

I am more satisfied with my life with my current medicine in comparison to my previous medication.

|  | Completely of the opposite opinion | Mostly of the opposite opinion | Somewhat of the opposite opinion | Neither of the opposite nor the same opinion | Somewhat of the same opinion | Mostly of the same opinion | Completely of the same opinion |
| --- | --- | --- | --- | --- | --- | --- | --- |
|  | ❏ | ❏ | ❏ | ❏ | ❏ | ❏ | ❏ |

Compared to my previous medication, my current medicine makes it easier to perform complex tasks (e.g. working).

|  | Completely of the opposite opinion | Mostly of the opposite opinion | Somewhat of the opposite opinion | Neither of the opposite nor the same opinion | Somewhat of the same opinion | Mostly of the same opinion | Completely of the same opinion |
| --- | --- | --- | --- | --- | --- | --- | --- |
|  | ❏ | ❏ | ❏ | ❏ | ❏ | ❏ | ❏ |

With my current medicine, my pain requires less of my attention in everyday life compared to when I have used the previous medication.

|  | Completely of the opposite opinion | Mostly of the opposite opinion | Somewhat of the opposite opinion | Neither of the opposite nor the same opinion | Somewhat of the same opinion | Mostly of the same opinion | Completely of the same opinion |
| --- | --- | --- | --- | --- | --- | --- | --- |
|  | ❏ | ❏ | ❏ | ❏ | ❏ | ❏ | ❏ |

My mood is better with the current medicine than it was with the previous medication.

|  | Completely of the opposite opinion | Mostly of the opposite opinion | Somewhat of the opposite opinion | Neither of the opposite nor the same opinion | Somewhat of the same opinion | Mostly of the same opinion | Completely of the same opinion |
| --- | --- | --- | --- | --- | --- | --- | --- |
|  | ❏ | ❏ | ❏ | ❏ | ❏ | ❏ | ❏ |

The current medicine takes the pain away more efficiently than the previous medication.

|  | Completely of the opposite opinion | Mostly of the opposite opinion | Somewhat of the opposite opinion | Neither of the opposite nor the same opinion | Somewhat of the same opinion | Mostly of the same opinion | Completely of the same opinion |
| --- | --- | --- | --- | --- | --- | --- | --- |
|  | ❏ | ❏ | ❏ | ❏ | ❏ | ❏ | ❏ |

Compared to my current medicine, my previous medication gave me more negative physical symptoms (e.g. nausea, tremors, headache, constipation).

|  | Completely of the opposite opinion | Mostly of the opposite opinion | Somewhat of the opposite opinion | Neither of the opposite nor the same opinion | Somewhat of the same opinion | Mostly of the same opinion | Completely of the same opinion |
| --- | --- | --- | --- | --- | --- | --- | --- |
|  | ❏ | ❏ | ❏ | ❏ | ❏ | ❏ | ❏ |

Compared to my current medicine, my previous medication gave me more negative psychological symptoms (e.g. tiredness, confusion, difficulties concentrating, difficulties with memory, mood problems).

|  | Completely of the opposite opinion | Mostly of the opposite opinion | Somewhat of the opposite opinion | Neither of the opposite nor the same opinion | Somewhat of the same opinion | Mostly of the same opinion | Completely of the same opinion |
| --- | --- | --- | --- | --- | --- | --- | --- |
|  | ❏ | ❏ | ❏ | ❏ | ❏ | ❏ | ❏ |

**Open question**

If you wish to, you can describe in your own words how your current medicine affects you. Please specify which medicine (medical opiates/opioids or medical cannabis) you mean.

|  |
| --- |

If you wish to, you can give us feedback/general comments!

|  |
| --- |
